# Feasibility Study for Finding Mathematical Approaches to Describe the Optimal Operation Point of Sensor-Based Sorting Machines for Plastic Waste

**DOI:** 10.3390/polym15214266

**Published:** 2023-10-30

**Authors:** Karl Friedrich, Nikolai Kuhn, Roland Pomberger, Gerald Koinig

**Affiliations:** 1 Mineral Processing, Department of Mineral Resources Engineering, Montanuniversitaet Leoben, Franz Josef-Strasse 18, 8700 Leoben, Austria; 2 Waste Processing Technology and Waste Management, Department of Environmental and Energy Process Engineering, Montanuniversitaet Leoben, Franz Josef-Strasse 18, 8700 Leoben, Austriaroland.pomberger@unileoben.ac.at (R.P.);

**Keywords:** sensor-based sorting, NIR sorting, optimal operation point, throughput rate, input composition, purity, recovery, regression model

## Abstract

At present, sensor-based sorting machines are usually not operated at the optimal operation point but are either overrun or underrun depending on the availability of waste streams. Mathematical approaches for predefined ideal mixtures can be found based on the input stream composition and the throughput rate. This scientific article compares whether and under what conditions these approaches can be applied to sensor-based sorting machines. Existing data for predefined ideal mixtures are compared with newly generated data of real waste on three sensor-based sorting setups in order to make significant statements. Five samples of 3D plastics at regular intervals were taken in a processing plant for refuse-derived fuels. With the comparison of all these results, four hypotheses were validated, related to whether the same mathematical approaches can be transferred from ideal mixtures to real waste and whether they can be transferred to sensor-based sorting machines individually or depending on the construction type. The developed mathematical approaches are regression models for finding the optimal operation point to achieve a specific sensor-based sorting result in terms of purity and recovery. For a plant operator, the main benefit of the findings of this scientific article is that purity could be increased by 20% without substantially adapting the sorting plant.

## 1. Introduction

Increasingly strict governmental guidelines for the efficiency of recycling plants and growing demands for recycling rates require state-of-the-art plant management to fulfil all obligations regarding the purity of product and tonnage of waste processed while still operating profitably. The new European Waste Framework Directive [1] requires a recycling rate for all municipal solid waste of 60% by 2030. Furthermore, in 2020, 34.6 kg per capita of plastic packaging waste had been generated per capita in the European Union with as little as 13.0 kg per capita being recycled [2]. Stricter legal requirements combined with the rising consumption of plastic packaging makes new innovative technologies necessary to increase the efficiency of existing waste-sorting plants to sort plastic waste and plastic from municipal solid waste.

One of the primary concerns regarding sorting plastic waste is the dichotomy of meeting both requirements, namely output with sufficient purity while maintaining a high yield and throughput rate to process sufficient amounts of plastic waste. The dichotomy lies in the effects of increasing throughput rate on sensor-based sorting (SBS) machines commonly used to sort plastic waste [3]. It has been shown that diminishing purity is expected with the increasing throughput rate [4].

Plastic waste can be sorted using optical sensors in SBS, triboelectrostatic forces or density separation with hydrocyclones [3]. However, the most applied technology for sorting post-consumer plastic waste is near-infrared (NIR) spectroscopy applying hyperspectral imaging [5]. NIR technology is a fast non-contact and non-destructive SBS method in waste management [6,7]. The NIR region covers a wavelength range between 750 to 2500 nm [8] and allows the differentiation of various materials based on the vibration of molecules excited by radiation. The emitted light leads to vibrational and rotational movements of molecules or parts of molecules of the material. As a result, the corresponding absorption bands can be captured with an optical sensor in form of a spectrum [9]. This spectrum provides information about the chemical composition of the sorting material and enables the detection of measurable separation properties of a material stream [10].

Two NIR sorting construction types are commonly used in waste processing plants, which differ in how the waste material is moved past the sensor. The movement is based either on gravity, when the material slides down a chute, or on mechanical forces using a conveyor belt. While granular material is usually sorted using a chute, bulky materials are sorted using conveyor belt machines [11]. The latter systems are also a focus of this study.

SBS plants are susceptible to changes in input quantity and quality (composition), with surface conditions significantly affecting sorting success [12] and plants being frequently overrun or underrun, reducing sorting efficiency [4].

Finding the optimal operation point for these SBS aggregates is paramount for the success and profitability of any waste processing plant. Empirical averages can perform this optimization and provide an acceptable approximation, but it is time-consuming and assumed to be repeated for every aggregate [4].

Developing a mathematical approach to optimize the throughput rate of a specific NIR sorting setup can save valuable time and increase the profitability of existing sorting setups. Moreover, it can reduce the energy amount for the process by operating the system at the optimal operation point.

## 2. Degree of Novelty and Industrial Relevance

Referring to the existing findings in the correlation of input composition, the throughput rate and the SBS results (purity, yield, recovery) from Küppers [4], we know that there is a correlation within these parameters in mathematical approaches for ideal self-composed plastic fractions with a defined mixing ratio (ideal mixtures).

The research novelty of this paper is to determine whether these mathematical approaches can be extended to real plastic waste and its input compositions because this is still unknown. Furthermore, when the same mathematical approach cannot be used for real waste, it is a point of interest how similar mathematical approaches would look for real plastic waste when the same sorting task is performed.

When a mathematical approach for real plastic waste can be developed, it raises the research question of what level of precision can be reached for a mathematical approach that covers input composition, throughput rate, SBS result purity, yield and recovery and, lastly, in which ranges or threshold values for these parameters the mathematical approach can work.

If a mathematical approach like that described above can be developed for real plastic waste, the waste treatment branch can be progressively improved. Friedrich [13] investigated this in an assessment of how sophisticated the waste-sorting industry is in using data analytics to improve their sorting processes.

The goal in waste-sorting plants is to achieve a required minimum threshold value for purity and to stay below threshold values for impurities, which differ between the several types of waste streams. These threshold values are regulated by law or the recycling process after sorting, e.g., for cullets in the container glass industry, the threshold values are regulated in Austria in the ”Quality requirements for cullets to be used in the container glass industry” guideline T 120 from Bundesverband Glas e.V. [14], while the threshold values for sorted plastic waste are defined in ”Quality standards for sorted plastic wastes for recycling” [15].

The main interest of a sorting plant operator is to achieve these qualities so that a recycling plant buys their produced sorted waste fraction but with a throughput rate as high as possible to have the maximum possible amount of waste treated and sold per year. Considering these facts, there is an optimal operation point for achieving a specific SBS result related to the input stream composition, throughput rate, purity, yield and recovery. For a plant operator, this means that purity could be increased by 20% without substantially adapting the sorting plant with the plant at optimal operation point.

The yield was not evaluated in this study since it is only relevant for optimization when the results in purity and recovery are sufficient. For this reason, this study is focused on purity and recovery.

For the mathematical approaches to find the optimum operation point, which is the result after the evaluation and processing of the created data, there are four hypotheses to be confirmed or negated in this study:

**Hypothesis** **1:**
*It is possible to create mathematical approaches for SBS machines, which mainly depend on the input composition of waste and the throughput rate.*

**Hypothesis** **2:**
*It is possible to create a generic mathematical approach for all SBS machines related to input composition, purity, recovery and throughput rate.*

**Hypothesis** **3:**
*It is possible to create a construction-type-specific (chute or belt sorter) mathematical approach for all SBS machines related to input composition, purity, recovery and throughput rate.*

**Hypothesis** **4:**
*It is possible to create a machine-specific mathematical approach for all individual SBS machines related to input composition, purity, recovery and throughput rate.*

## 3. Materials and Methods

Sorting efficiency is commonly analyzed based on recovery (R), yield (R_W_) and purity (P_m_), three mass-specific (m%) indicators. These were previously reported by Friedrich [10], as defined in the following paragraphs.

Recovery (R) is the quotient of product mass or mass of ejected material (m_eject_) and total mass of input (m_input_) over a given period. Recovery indicates the product produced per unit of time or a given throughput rate.

$$R=\frac{m_{eject}\left[\frac{t}{h}\right]}{m_{input}\left[\frac{t}{h}\right]}\times 100\%$$

Yield (R_w_) is defined by the quotient of the product produced in the output (m_eject_ × c_eject_) and valuable materials in the input (m_input_ × c_input_). With the mass flow of the output material (m_eject_) and the calculated recyclable material concentration in the output fraction (c_eject_), the quantity of valuable material generated in the output is calculated.

$$R_{w}=\frac{m_{eject}\left[\frac{t}{h}\right]\times c_{eject}[\%]}{m_{input}\left[\frac{t}{h}\right]\times c_{input}[\%]}\times 100\%$$

Feil proposed a further quality indicator with the calculation of purity P_m_ [16]. The percentage of correctly ejected input material—purity—is calculated as follows.

$$P_{m}=\frac{m_{\mathrm{recyclable material}}[\frac{t}{h}]}{m_{impurity}\left[\frac{t}{h}\right]+m_{\mathrm{recyclable material}}[\frac{t}{h}]}\times 100\%$$

### 3.1. Materials

Since there are many different plastic types, the first step is to define a plastic waste stream which is quite similar to the ideal materials Küppers [4] used. As these were washed and dried polyolefin plastic flakes, it was decided to use refuse-derived fuel (RDF) sampled in a waste treatment plant where an SBS machine can be installed to sort mixed plastic waste into separate plastic fractions.

The next step was to repeat the trials of Küppers [4] on the same experimental SBS setup to determine the similarity of the mathematical approaches depending on the plastic waste stream. Then, RDF trials were further performed on two SBS setups with different construction types to evaluate whether the found mathematical approaches are generally valid for SBS setups, depending on the construction type or individually on the SBS setup. In the end, the optimal operation point for achieving a specific sensor-based sorting result could be described with mathematical approaches. The material was not further processed after the sampling; it was used directly in the sampled condition for the trials.

### 3.2. SBS Setups

The trials to be compared were performed on three different SBS setups, the experimental SBS setup at Montanuniversitaet Leoben, the sensor-based chute sorting setup for technical facilities from an SBS machine provider and the sensor-based belt sorting setup for technical facilities from another SBS machine provider. All setups are designed as two-way systems for SBS.

**Figure 1:**
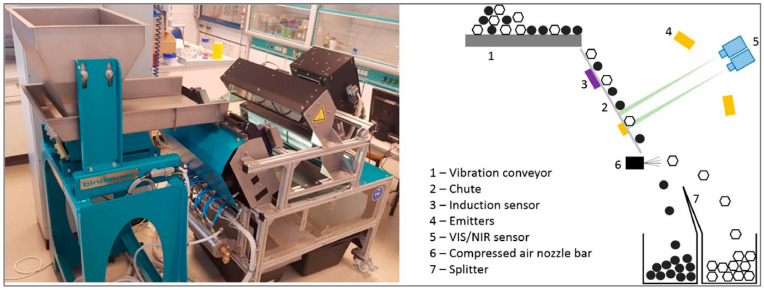
Experimental SBS setup at Montanuniversitaet Leoben [4].

The basic concept of a two-way system can be seen in Figure 1: The input fraction is passed through a vibration conveyor (1) to either a chute or a belt (2), an optional induction sensor (3) to detect metals, emitters (4) to detect the particles with signals from imaging sensors (5) and a compressed air nozzle bar (6), which separates the particles into an eject and a reject fraction. Both output boxes are separated through a splitter (7).

1. Experimental SBS setup

The experimental SBS setup is located at the Chair for Waste Processing Technology and Waste Management at Montanuniversitaet Leoben. It is a chute sorter manufactured with an open design to allow simple conversions to be carried out for deriving process influences. The setup is equipped with three common sensors for waste sorting (Figure 1) [10]:

- An NIR sensor to determine the molecular composition of NIR active particles;
- A color line scan camera to determine the visible light absorbance of elementary components used for sorting by color;
- An induction sensor to identify metallic components/particles.

The present research uses the NIR sensor Helios NIR G2-320 provided by EVK DI Kerschhaggl GmbH, Raaba, Austria [17]. The chute width of the system is 0.5 m. Further details of the setup can be found in [11].

2. Sensor-based chute sorting setup for technical facilities

**Figure 2:**
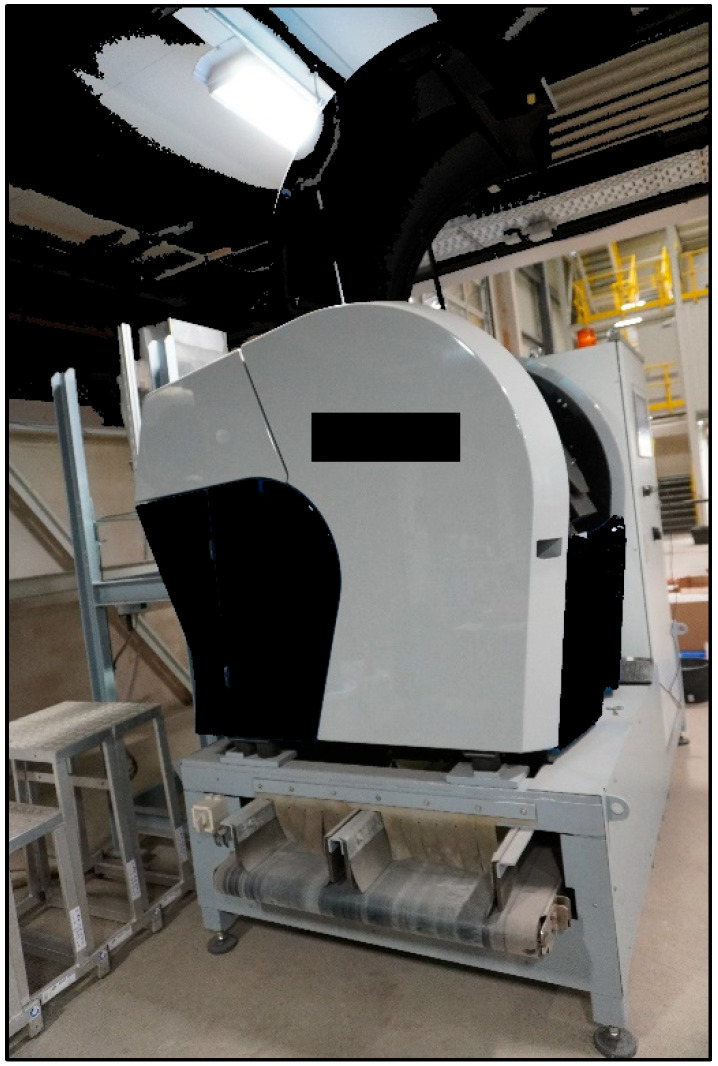
Sensor-based chute sorting setup for technical facilities (own depiction).

The provider of the used sensor-based chute sorting setup (Figure 2) has a technical facility where the trials are conducted. It is designed as a two-way system, which can be set up with different sensors like visual spectroscopy (VIS) transmission/reflection, NIR and metal detection. In the present feasibility study, the NIR sensor Photonfocus MV3-D640I-CL was used. The system is endowed with a chute width of 0.4 m.

3. Sensor-based belt sorting setup for technical facilities

**Figure 3:**
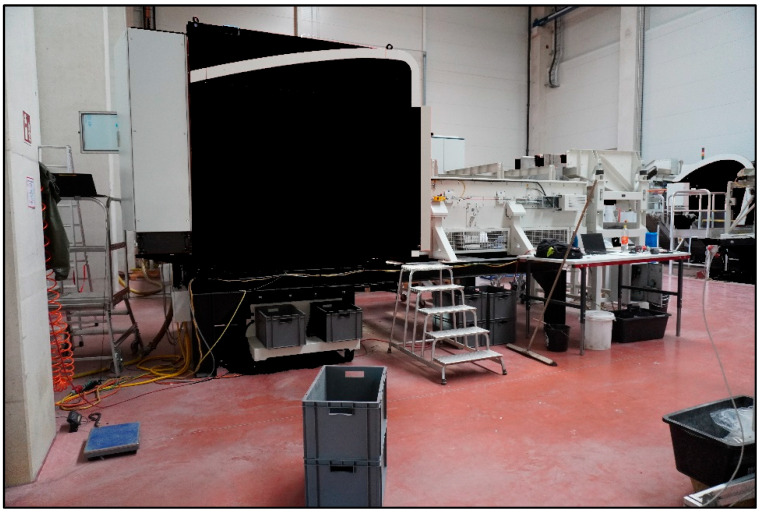
Sensor-based belt sorting setup for technical facilities (own depiction).

The sensor-based belt sorting setup used (Figure 3) was prepared by another provider with a technical facility where the trials were conducted. The machine was built up as a two-way machine and can be set up with different sensors like an RGB line scan camera for VIS sorting, an NIR sensor and a metal detection sensor. The NIR sensor Inno-Spec RedEye 1.7 was used in the present feasibility study. The system is endowed with a belt width of 1.2 m, although the belt was split in the middle for the trials so that the working width for the trials was 0.6 m.

Table 1 overviews the different NIR sensors installed in the used SBS systems: Helios NIR G2-320 [17], Photonfocus MV3-D640I-CL [18] and Inno-Spec RedEye 1.7 [19].

The statistical evaluation software of the identification result works on each of the SBS setups in the same way. All of them prepare the pixel statistic, the material statistic and the object statistic. These values are used to calculate the purity of the sorted fractions to be compared for the SBS setups.

### 3.3. Methods

The methodology and the experimental design to confirm or negate the four hypotheses are structured as follows:

1. Phase

The first phase was developing an NIR sorting model to compare the SBS setup performance. Sample 1 was used as a reference sample (“sorting model creation sample”) to record the raw spectra to be taught and is not included in the trials. This procedure was chosen to have the material classes of the creation samples available as identical fractions for further test series on all used sensor-based sorting setups for comparability.

In order to ensure comparability, the same raw spectra from PET and PP, which were published by Küppers [4], were used in the NIR sorting model for the RDF fraction on the experimental SBS setup. Further spectra must be recorded first in order to be added to the NIR sorting model.

Using the same raw spectra files for the sensor-based chute sorting setup and the sensor-based belt sorting setup is impossible since the software does not allow the files to be imported from the experimental SBS setup. In these setups, all of the raw spectra have to be newly recorded and new NIR sorting models developed.

2. Phase

The second phase was performing trials with four RDF fractions with at least three different throughput rates to obtain mathematical approaches for each SBS setup. In sum, at least 108 trials were performed. The number of further trials depends on each setup’s time availability and the recorded data’s plausibility.

3. Phase

Expected results were predicted with regression algorithms in machine learning. The input parameters were used to predict the respective output parameters using regression. Regression helps define the relationship between the target variable to be predicted and created data points. It is a type of supervised learning in machine learning that helps map a predictive relationship between target values and data points. The regressions used in this study are gaussian process regression (GPR) and a regression neuronal network (RNN) for finding the best-fitting regression model in a mathematical approach. The input parameters (regressors) are the input composition and the throughput rate, the output parameters (regressands) are purity and recovery.

MATLAB code was developed to evaluate the trial results in regression models and principal component analysis for finding mathematical approaches. All computation was carried out using MATLAB by MathWorks (Natick, MA, USA) using “9.13.0.2105380 (R2022b) Update 2” on a Windows 10 computer equipped with an Intel^®^ UHD Graphics 630 GPU and an Intel ^®^ Core ™ i5-9400H CPU clocked at 2.50 GHz.

A ranking of features for regression using the MRMR algorithm was performed to determine which of the following parameters influence the sorting result:

- Input composition based on the purity of the input fraction: amount of target material to be ejected of the input fraction (m%);
- Throughput rate: amount of mass per hour through the sorting setup (kg/h);
- Target material: material class, which is ejected, depending on the NIR sorting model (-);
- Aggregate: type of SBS setup to be used (-).

MRMR algorithms were used to find an optimal set of features that is dissimilar and represents the response variable effectively. The parameters listed above were evaluated according to their influence in the sorting result in a predictor ranking with a predictor importance score.

Then, a statistical evaluation was carried out with RMSE and R^2^; the regressions were performed with GPR and the RNN. The results are shown in 3D and 2D diagrams depending on which fits better for visualization.

The models, GPR and the RNN, were trained on a training set comprising 70% of the data. The testing set consisted of the remaining 30% of the collected data in compliance with existing findings depicting the ideal training/test split. The statistical values mentioned above were calculated from the model’s performance on the test set. A short introduction to the underlying methods of GPR and the RNN is given in the following section.

The task at hand can be defined as a regression task. This initial distinction is necessary to evaluate the feasible machine learning tools. Regression is a type of supervised machine learning task where the goal is to predict a continuous numeric output (a real-valued number) based on input data. Supervised machine learning implies that the prediction algorithm is trained on labeled data consisting of input–output pairs (X, y), where X represents the input data, and y represents the corresponding output values. The model aims to learn to capture the underlying relationships.

A GPR (gaussian process regression) model is a probabilistic, non-parametric machine learning algorithm used for regression tasks. It is a powerful tool for modeling and predicting continuous data, particularly when dealing with uncertainty. GPR is based on the principles of gaussian processes, which are a method of obtaining probability distributions over functions. GPR as a non-parametric model is well suited for the underlying data as it does not make strong assumptions about the functional form of the data. This is a stark contrast to parametric models like linear regression, which assumes a fixed functional form (e.g., a linear relationship), As such, GPR can capture a wide range of functions by learning from data.

Regression neural networks (RNNs) are a type of artificial neural network (ANN) specifically designed for solving regression problems. RNNs are powerful tools for modeling complex relationships between input features and continuous target variables. RNNs consist of multiple layers of interconnected neurons, organized into an input layer, one or more hidden layers and an output layer. Each neuron is associated with a weight and a bias, which are adjusted during training to optimize the network’s predictive capability.

4. Phase

The found regression models were used to test the four hypotheses of this study regarding finding an SBS machine’s optimal operation point. The hypotheses were confirmed or negated. Each confirmed hypothesis can help in finding the optimal operation point and describing it depending on the result and the number of confirmed hypotheses.

## 4. Results and Discussion

The investigated materials were sampled in an RDF processing plant. The plant processes 16 to 17 t/a input waste streams. These waste streams consist of 70 to 80% residue from sorting processes in several waste-sorting plants for lightweight packaging waste. Of this waste, 20 to 30% comprises high caloric fractions. The waste is comes from commercial waste collection. The output fractions of the plant are 2D and 3D RDF fractions.

**Figure 4:**
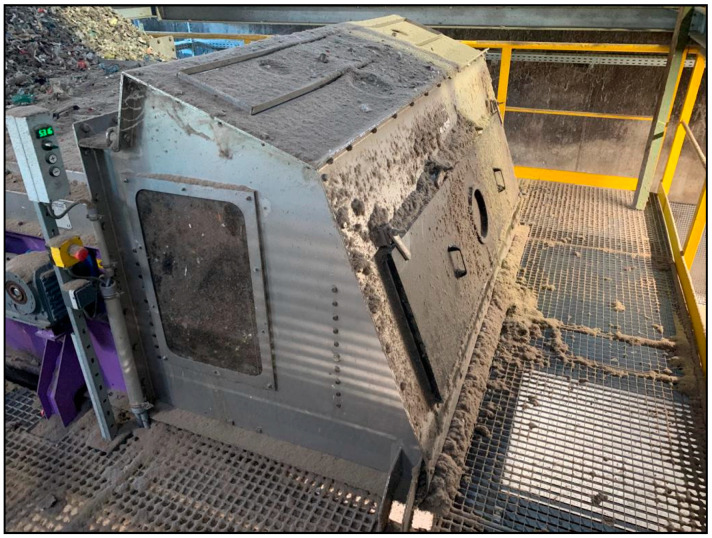
Second centrifugal separator in the RDF processing plant (own depiction).

After PVC and metal separation of the mixed plastic fractions, the fraction was shredded to a grain size > 100 mm. Next, the material was passed through a fluted screen before a two-stage centrifugal separator was used to obtain 2D and 3D RDF fractions. The fraction relevant to this work is the 3D RDF fraction. For this reason, the material for this paper was sampled after the second centrifugal separator (Figure 4).

For sampling, the flap at the side of the second centrifugal separator was opened, and the samples were taken during the fall of the conveyor belt. Since the samples must be representative to indicate the material stream, the sampling was carried out with a bucket moving slowly from left to right at the fall point of the conveyor belt. This procedure was performed five times over several days so that the five samples represented the variability of the RDF processing plants’ waste streams after the second centrifugal separator.

**Figure 5:**
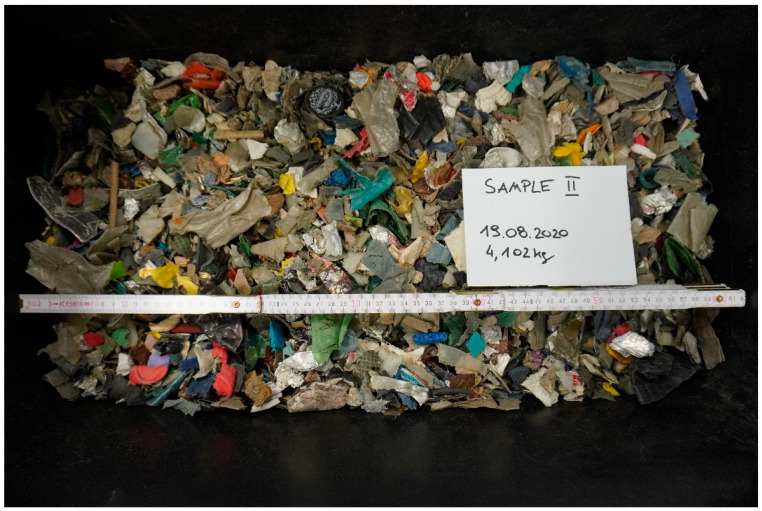
Sample 2, sampled on 19 August 2020, mass 4.102 kg (own depiction).

Sample 1 from 17 August 2020 was used as the reference fraction for creating an NIR sorting model of the five samples taken. Sample 1 was chosen because it has a higher mass than the other four samples and thus represents a larger range of particles for the sorting model creation. Sample 2 is presented in Figure 5 as an example of how the sorted samples look.

In the first step, the sampled RDF fractions must be prepared before starting the trials. Metal particles were separated from all fractions with the induction sensor of the experimental SBS setup at the Chair of Waste Processing Technology and Waste Management of Montanuniversitaet Leoben. Metals have a higher density than plastics in the RDF fraction, which requires different setup parameters due to the delay time for the material discharge or the discharge pressure setting. Furthermore, Furthermore, the SBS is installed in plants after the metal separation in plants after the metal separation, which means the mathematical approaches may be used with a material flow already decontaminated from metal. Table 2 shows the sample composition of metals and metal-free fractions in their mass and percentage distribution.

Next, the components of the metal-free fractions were analyzed in detail based on visual inspection followed by NIR characterization on the experimental SBS setup at Montanuniversitaet Leoben. For the characterization, the NIR sorting model used by Küppers [4] was applied for PET, PE and PP and extended with further new material classes, as shown in Table 3. Materials not listed in Table 3, such as textiles, are included in the rest “MC” (material class) category. A further breakdown of the classes is not required since individual large particles are responsible for the high mass fraction. Furthermore, particles could not be assigned to increased fine grain fraction (>2 mm), or the masses were too low to assign them to another new fraction.

For the trials, PET, PP and PVC were considered due to their abundance in the waste composition (Table 3). LDPE consists of many small 2D films, for which sorting is not effectively possible with the same machine settings as for 3D plastics due to their ability to fly and low density. PVC was considered because it is not desirable in RDF due to its chlorine content and must be discharged anyway. All other material classes were summed up as the Rest category. Table 4 shows the material classes considered for this study.

After each trial, the results included the calculated purity and recovery. Next, the four hypotheses developed in this study were either confirmed or negated. In the end, the industrial outreach of the findings was predicted.

**Hypothesis** **1:**
*It is possible to create mathematical approaches for SBS machines, which mainly depend on the input composition of waste and the throughput rate.*

The input parameters of each sorting trial were the waste stream’s input composition and the sorting process’s throughput rate. A feature evaluation was carried out to ascertain the influence on the sorting results of the parameters input composition (based on the purity of the input fraction), throughput rate, target material to be sorted and aggregate (SBS setup).

Intuitively, one would expect the input composition and throughput rate to be the dominant input variables for predicting the output’s purity and the recovery of valuable materials. This is because neither the target material nor aggregate change in response to the input material and thus only allow for a rough estimate of achievable purity and recovery.

**Figure 6:**
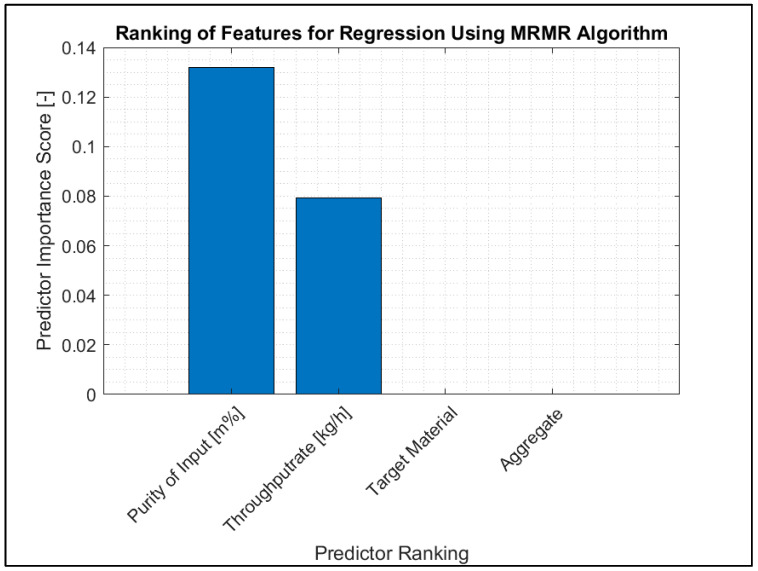
Ranking of features for regression using MRMR algorithm.

The performed feature selection supports this intuition. The results of ranking features for regression using MRMR algorithms (Figure 6) show that two of the four evaluated parameters mainly influence the sorting result. The target material to be sorted and the selected aggregate (SBS setup) have low prediction value. This means the sorting result mainly depends on the purity of the input material and the throughput rate. The purity of the input has a predictor importance score of 0.13, while the throughput rate has a score of 0.08.

The performed analysis of models trained on all four input variables in comparison to models trained on input composition and throughput further supports this. Models trained on all available input variables performed as well as or worse than their counterparts that adhered to the MRMR feature selection.

Table 5 shows the results of the preliminary sensitivity analysis to gauge the model response to the inclusion of aggregate type and target material in addition to input composition and throughput. The gain is minute as the model performance on the testing set did not improve when including aggregate type and target material in the training data set.

In consideration of Figure 6, **Hypothesis 1 can be confirmed.**

**Hypothesis** **2:**
*It is possible to create a generic mathematical approach for all SBS machines related to input composition, purity, recovery and throughput rate.*

In order to find a mathematical approach which describes the relation between input composition, throughput rate, purity and recovery for all trials, these data is used to create regression models.

**Figure 7:**
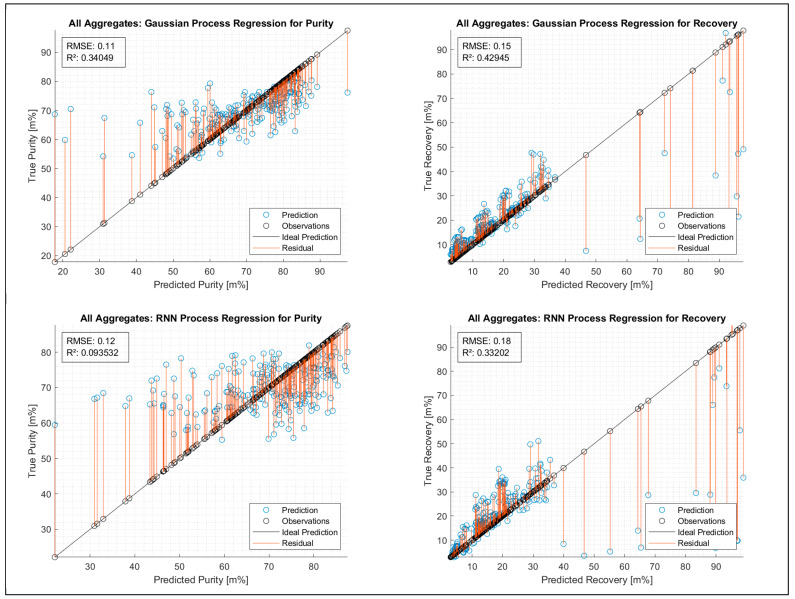
GPR and RNN process regressions for purity and recovery for all aggregates (SBS setups).

Figure 7 shows the deviation of predicted and observed values for purity and recovery in regression models. An ideal prediction of the sorting result related to purity or recovery was considered a low deviation between prediction and observation.

For purity, both of the regressions—GPR and RNN—do not allow the development of a mathematical approach that might work for a significant prediction. The RMSE with 11 and 12% and the R^2^ with 0.34 and 0.094 indicate that a mathematical approach related to the input composition and the throughput rate, valid for all SBS setups, cannot be developed.

For recovery, the regression models work well up to 40 m%; from then on, only sporadic data points are available. The model works in the areas depicted by the data, as expected. The values for RMSE with 15 and 18% are far too low, as is the R^2^ of 0.42945 and 0.33202, to develop the desired mathematical approach.

In consideration of Figure 7, **Hypothesis 2 can be negated.**

**Hypothesis** **3:**
*It is possible to create a construction-type-specific (chute or belt sorter) mathematical approach for all SBS machines related to input composition, purity, recovery and throughput rate.*

According to the result for all aggregates in Figure 7, it can be predicted that a mathematical approach which describes purity and recovery related to input composition and 439 throughput rate machine type-specific cannot be developed. The deviations between the 440 prediction and the observations are severe—as described in the previous hypothesis 2—441 to get suitable mathematical approaches for predicting sorting results.

In consideration of Figure 7, **Hypothesis 3 can be negated.**

**Hypothesis** **4:**
*It is possible to create a machine-specific mathematical approach for all individual SBS machines related to input composition, purity, recovery and throughput rate.*

For the data of trials with ideal mixtures on the experimental SBS setup, the RNN developed a better regression model. For the RDF trials on the experimental SBS setup, the sensor-based chute sorting setup and the sensor-based belt sorting setup for technical facilities, GPR developed the superior model.

According to the analysis of the experimental SBS setup data, the regression models differ from the type of waste to be sorted for purity and recovery. This means it is not the target material itself that influences the sorting result, as proven in hypothesis 1, but rather the condition of the waste type to be sorted. The ideal mixture was created from plastic packaging waste shredded to a grain size >30 mm, while the RDF was shredded to a grain size >100 mm. It can be stated that the pretreatment and condition of the waste influence the regression models.

The regression models on the used SBS setups for RDF differ from setup to setup, although the material is the same. This indicates that when regression models are used to describe the correlation between the input and output parameters of a sorting process, these models have to be created separately for each SBS setup.

**Figure 8:**
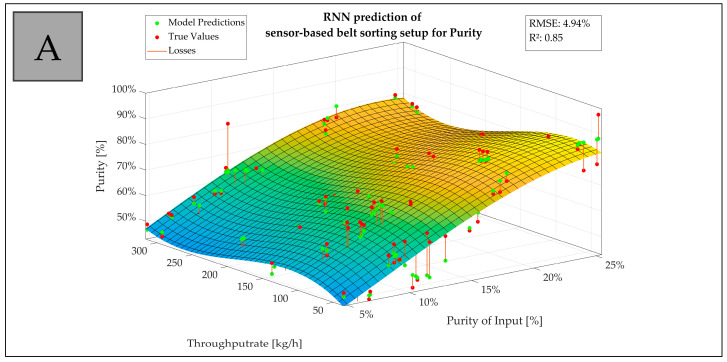

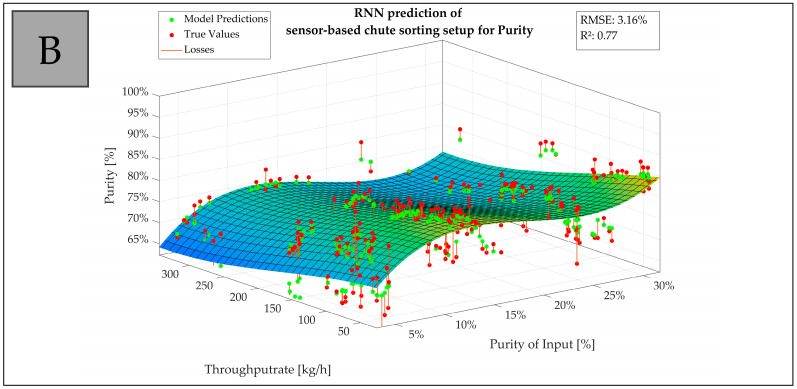
Example depiction of model predictions on test sets with losses calculated on model prediction vs. true value with a 3D fitted curve: trend of the actual data related to the purity of input and throughput rate on the sensor-based belt sorting setup (**A**) and the sensor-based chute sorting setup (**B**) including R^2^ and RMSE for the underlying prediction model.

Figure 8 visualizes an example depiction of model predictions on test sets with losses calculated on model prediction vs. true value with a 3D fitted curve. It shows the trend of the actual data related to the purity of input and throughput rate on the sensor-based belt sorting setup (A) and the sensor-based chute sorting setup (B) including R^2^ and RMSE for the underlying prediction model.

For interpreting the findings of each SBS setup, a statistical evaluation of the regression models was carried out with RMSE and R^2^. The results are listed in Table 6.

For purity, the values for R^2^ are between 0.50306 for GPR with RDF on the experimental SBS setup and 0.87458 for GPR with RDF on the sensor-based belt sorting setup, which is a difference that does not allow a general statement. What needs to be considered is that the experimental SBS setup was designed for analyzing and sorting flakes, which led to a higher scattering of the output data and deteriorated the regression model. The RMSE was between 3% for GPR with RDF on the sensor-based chute sorting setup and 7% for the RNN with ideal mixtures on the experimental SBS setup, which is distinctly good.

The R^2^ for the recovery regression models varies between 0.92686 for GPR with RDF on the sensor-based chute sorting setup and 0.96956 for GPR with RDF on the experimental SBS setup. RMSE resides between 1% for GPR with RDF on the sensor-based belt sorting setup and 3% for the RNN with ideal mixtures on the experimental SBS setup. This means that the mean variation is low, and the regression models suitably describe the recovery result.

Comparing the regression models, excluding the experimental SBS setup with RDF, the models describe the sorting result behavior regarding the input parameters (input composition, throughput rate) sufficient to regulate an SBS setup when a specific sorting result in terms of purity and recovery can be achieved.

Considering Figure 8 and Table 6, **Hypothesis 4 can be confirmed for the scope of applied data.**

### Industrial Outreach

The added value for the industrial waste-sorting plants can be derived from the outcome of the four hypotheses. The optimal operation point is the maximum possible throughput rate with the expected purity of sorted waste. In short, the sorted waste mass with an expected result is maximized for a specific time frame.

The regression models for purity and recovery make it possible to predict a sorting result with the knowledge of the input composition and throughput rate with the calculated deviation range. This in turn means that the sorting result should be a specific expected purity. The regression models deliver the maximum possible throughput rate (within the model deviation range) to reach the postulated purity.

**Figure 9:**
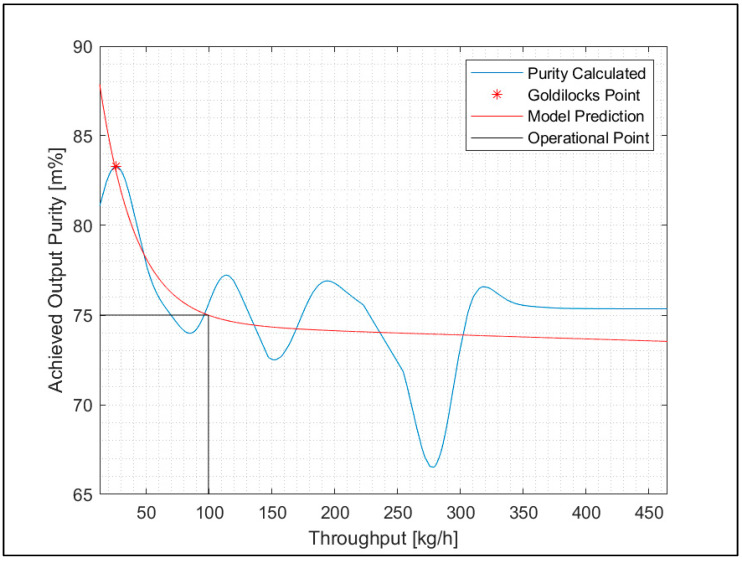
“Goldilocks point” and optimal operation point with RNN regression related to a purity of 75% and throughput rate of 100 kg/h of the experimental SBS setup.

An explanation is shown in Figure 9. When a purity of 75% is required, an RNN regression model for the experimental SBS setup with RDF can reach a maximum throughput rate of 100 kg/h.

Furthermore, Figure 9 shows the Goldilocks point for the regression model. This principle appears when there is a clear optimum for a value. The Goldilocks point is reached when the highest calculated purity hits the model’s predicted purity for the maximum possible throughput rate. As the Goldilocks point for that model can only be reached for an unacceptably low throughput rate, the scenario in Figure 9 is not realistic for running a plant.

Another option for using the regression models is to simulate circuit operation in sorting plants or stepwise sorting with more SBS machines. The idea is to utilize a first sorting step to enrich concentrates of a specific plastic type and sort them into recyclates in a second or third sorting step. For example, in Figure 9 the first sorting step achieves a purity of 75%; with the second sorting step, the concentration of the target material starts at 75% so that a higher purity can be achieved with the same throughput rate. This principle can be used especially in small sorting plants, which can run circuit operation, or in larger sorting plants, which have either the opportunity for circuit operation or have more sorting machines that run stepwise in sequence.

Using these findings allows for a sorting plant to increase purity by running the plant on the optimal operation point without substantially adapting the plant. The only requirement is to create regression models with recorded production data to find the optimal operation point for several waste input compositions of the sorting process. Input compositions can easily be recorded by installing an NIR input characterization before the SBS machine with the same NIR sorting model selected on the SBS machine.

## 5. Conclusions

Sorting plant operators want to achieve specific levels of recyclate purity so that a recycling plant buys their produced sorted waste fraction, but with the maximum possible throughput to ensure the maximum possible amount of waste treated and sold within a year. This leads to an interest in finding the optimal operation point for achieving a specific SBS result related to the input stream composition, the throughput rate, the purity and the recovery.

The research task of this study was to find mathematical approaches in regression models which cover input composition, throughput rate, the SBS results in purity and recovery and, lastly, in which ranges or threshold values these parameters can be used. For the regression models, with the results of 108 trials on three sensor-based sorting setups, with ideal mixtures and five RDF samples, four hypotheses were confirmed or negated in this study.

**Hypothesis** **1:**
*It is possible to create mathematical approaches for SBS machines, which mainly depend on the input composition of waste and the throughput rate.*

This hypothesis is confirmed. The sorting result mainly depends on the purity of the input, which means the amount of target material to be sorted consists of the input fraction. Furthermore, according to the feature ranking, the type of material sorted has a weak influence on the result.

**Hypothesis** **2:**
*It is possible to create a generic mathematical approach for all SBS machines related to input composition, purity, recovery and throughput rate.*

Developing a regression model that works for a significant sorting result prediction is not feasible. The statistical results indicate that developing a mathematical approach related to the input composition and the throughput rate, valid for every SBS setup, is impossible.

**Hypothesis** **3:**
*It is possible to create a construction-type-specific (chute or belt sorter) mathematical approach for all SBS machines related to input composition, purity, recovery and throughput rate.*

This hypothesis is negated according to the results of hypothesis 2.

**Hypothesis** **4:**
*It is possible to create a machine-specific mathematical approach for all individual SBS machines related to input composition, purity, recovery and throughput rate.*

The regression models indicate that the sorting result behavior regarding the input parameters (input composition, throughput rate) is sufficient in their scope of applied data to regulate an SBS setup when a specific sorting result in terms of purity should be achieved. Furthermore, the waste pretreatment or condition before the SBS machine influences the sorting result. The findings of this scientific article demonstrate that regression models with a sufficient number trials to ensure an acceptable prediction accuracy in RMSE and R^2^ can enable a sorting plant to automatically regulate its throughput to achieve a specific purity in the sorting process.

This leads to automatic plant operation and maximizes the mass sorted in the plant with expected purity. The only requirement is the installation of an NIR input characterization device before the SBS machine to determine the input composition and to record the production data. Furthermore, the regression models can also simulate circuit operation in sorting plants or stepwise sorting with more SBS machines. After the first sorting step to enrich concentrates of a specific plastic type, the recyclates are sorted in a second or third sorting step.

In summary, the findings of this scientific article can be utilized to enable a sorting plant to increase purity by running at the optimal operation point without substantial adaptation. Superordinate considered, this helps to increase the amount of recycled plastic so that less plastic waste is thermally treated without substantial investments in adapting current plant designs.

## Figures and Tables

**Table 1:** NIR sensor specification overview of the different types installed in the used SBS systems [17,18,19].

Sensor-Based Sorting Setup	Experimental Sensor-Based Sorting Setup	Sensor-Based Chute Sorting Setup for Technical Facilities	Sensor-Based Belt Sorting Setup for Technical Facilities
NIR Sensor	Helios NIR G2-320	Photonfocus MV3-D640I-CL	Inno-Spec RedEye 1.7
Technology	n.a.	Ingas with CMOS read out circuit	InGaAs
Resolution	n.a.	649 × 512	320 × 256
Pixel size	30 µm × 30 μm	15 µm × 15 µm	30 µm × 30 μm
Spectral range	930 to 1700 nm	930 to 1700 nm	950 to 1700 nm
Line scan rate	500 Hz full frame	n.a.	n.a.
Spectral resolution	9 nm	n.a.	9 nm
Spectral sampling	3.1 nm	n.a.	n.a.
Spatial resolution	312 pixels	n.a.	rms spot radius < 35 µm
Slit width	100 µm	n.a.	80 µm
Frame rate	n.a.	300 fps	330 fps

**Table 2:** Sample composition divided into metals and metal-free fractions.

Sample	1		2		3		4		5	
Sampling Date	17.08.2020		19.08.2020		02.10.2020		06.10.2020		20.10.2020	
Unit	(kg)	(m%)	(kg)	(m%)	(kg)	(m%)	(kg)	(m%)	(kg)	(m%)
Metal-free	6.33	86.8	2.88	70.3	3.88	74.3	3.82	85.4	3.86	90.0
Metal	0.96	13.2	1.22	29.7	1.34	25.7	0.66	14.6	0.43	10.0
Sum	7.29	100.0	4.10	100.0	5.22	100.0	4.48	100.0	4.29	100.0

**Table 3:** Sample composition of the metal-free fraction from selected individual fractions considered.

Sample	1		2		3		4		5	
Sampling Date	17.08.2020		19.08.2020		02.10.2020		06.10.2020		20.10.2020	
Unit	(kg)	(m%)	(kg)	(m%)	(kg)	(m%)	(kg)	(m%)	(kg)	(m%)
PP	0.94	14.9	0.38	13.3	0.46	11.8	0.46	12.1	0.44	11.5
PET	1.68	26.5	0.73	25.2	0.85	22.0	1.17	30.5	1.23	32.0
PVC	0.25	3.9	0.14	4.9	0.26	6.8	0.17	4.4	0.11	3.0
Wood/Foam	0.97	15.3	0.43	14.8	0.94	24.2	0.70	18.4	0.70	18.0
TPU	0.06	1.0	0.01	0.3	0.02	0.4	0.02	0.4	0.01	0.3
HDPE	0.18	2.8	0.06	2.0	0.05	1.2	0.04	1.0	0.11	2.9
LDPE	0.52	8.2	0.30	10.4	0.41	10.5	0.45	11.7	0.55	14.2
PS	1.00	15.8	0.72	25.0	0.64	16.4	0.54	14.3	0.57	14.8
Rest “MC“	0.74	11.6	0.12	4.1	0.26	6.7	0.28	7.2	0.13	3.2
Sum	6.33	100.0	2.88	100.0	3.88	100.0	3.82	100.0	3.86	100.0

**Table 4:** Sample composition used for the trials.

Sample	1		2		3		4		5	
Sampling Date	17.08.2020		19.08.2020		02.10.2020		06.10.2020		20.10.2020	
Unit	(kg)	(m%)	(kg)	(m%)	(kg)	(m%)	(kg)	(m%)	(kg)	(m%)
PP	0.94	14.9	0.38	13.3	0.46	11.8	0.46	12.1	0.44	11.5
PET	1.68	26.5	0.73	25.2	0.85	22.0	1.17	30.5	1.23	32.0
PVC	0.25	3.9	0.14	4.9	0.26	6.8	0.17	4.4	0.11	3.0
Rest	3.46	54.7	1.63	56.6	2.31	59.5	2.03	53.0	2.07	53.5
Sum	6.33	100.0	2.88	100.0	3.88	100.0	3.82	100.0	3.86	100.0

**Table 5:** Results of input variable sensitivity analysis for all aggregates using two or four of the available input variables.

Model	R^2^ (-)		RMSE (%)		Target
Used Parameters	2/4	4/4	2/4	4/4	
RNN for Purity	0.29	0.22	10	11	Purity
GPR for Purity	0.57	0.59	8	8	Purity
RNN for Recovery	0.48	0.5	15	15	Recovery
GPR for Recovery	0.59	0.48	14	15	Recovery

**Table 6:** Statistical evaluation results of the regression models with RMSE and R^2^.

SBS Setup	Sample	Regression Model	Purity		Recovery	
Statistical Value			RMSE (%)	R^2^ (-)	RMSE (%)	R^2^ (-)
Experimental SBS setup	Ideal mixtures	RNN	7	0.84921	3	0.95786
Experimental SBS setup	RDF	GPR	5	0.50306	2	0.96956
Sensor-based chute sorting setup	RDF	GPR	3	0.75158	2	0.92686
Sensor-based belt sorting setup	RDF	GPR	5	0.87458	1	0.95461

## Data Availability

The data presented in this study are available on request from the corresponding author.
